# Effects of Fat and Carnitine on the Expression of Carnitine Acetyltransferase and Enoyl-CoA Hydratase Short-Chain 1 in the Liver of Juvenile GIFT (*Oreochromis niloticus*)

**DOI:** 10.3390/genes15040480

**Published:** 2024-04-10

**Authors:** Ruijie Guo, Kai Huang, Kai Yu, Jinghua Li, Jiao Huang, Dandan Wang, Yuda Li

**Affiliations:** 1 College of Animal Science and Technology, Guangxi University, Nanning 530004, China; 2118401008@st.gxu.edu.cn (R.G.); yukai@st.gxu.edu.cn (K.Y.); 13152642320@163.com (J.H.); 18282584155@163.com (D.W.); 17508925370@163.com (Y.L.); 2 Fisheries Research and Technology Extension Center of Shaanxi, Xi’an 710086, China; derogbarepublic@163.com

**Keywords:** GIFT, carnitine acetyltransferase, enoyl-CoA hydratase short-chain 1, fat, carnitine

## Abstract

Carnitine acetyltransferase (CAT) and Enoyl-CoA hydratase short-chain 1 (ECHS1) are considered key enzymes that regulate the β-oxidation of fatty acids. However, very few studies have investigated their full length and expression in genetically improved farmed tilapia (GIFT, *Oreochromis niloticus*), an important aquaculture species in China. Here, we cloned CAT and ECHS1 full-length cDNA via the rapid amplification of cDNA ends, and the expressions of CAT and ECHS1 in the liver of juvenile GIFT were detected in different fat and carnitine diets, as were the changes in the lipometabolic enzymes and serum biochemical indexes of juvenile GIFT in diets with different fat and carnitine levels. CAT cDNA possesses an open reading frame (ORF) of 2167 bp and encodes 461 amino acids, and the ECHS1 cDNA sequence is 1354 bp in full length, the ORF of which encodes a peptide of 391 amino acids. We found that juvenile GIFT had higher lipometabolic enzyme activity and lower blood CHOL, TG, HDL-C, and LDL-C contents when the dietary fat level was 2% or 6% and when the carnitine level was 500 mg/kg. We also found that the expression of ECHS1 and CAT genes in the liver of juvenile GIFT can be promoted by a 500 mg/kg carnitine level and 6% fat level feeding. These results suggested that CAT and ECHS1 may participate in regulating lipid metabolism, and when 2% or 6% fat and 500 mg/kg carnitine are added to the feed, it is the most beneficial to the liver and lipid metabolism of juvenile GIFT. Our results may provide a theoretical basis for GIFT feeding and treating fatty liver disease.

## 1. Introduction

With the rapid development of intensive fish farming in recent years, various diseases caused by diet and environmental factors have become increasingly problematic [1]. A high-fat diet is a current trend in aquaculture, which might help to improve growth and reduce the increasing cost of protein ingredients and the limited supply of fish meals worldwide [2,3]. However, overfeeding and dietary nutrient imbalance often induce lipid deposition and lead to a significant accumulation of fatty acids in the liver of fish, which may induce fatty liver disease, one of the most common diseases in farmed fish [4,5,6]. Genetically improved farmed tilapia (GIFT, *O. niloticus*) is an excellent growth and adaptable species in southern China, such as Hainan, Guangxi, Guangdong, and Fujian [7,8]. Tilapia with hepatic steatosis grow slowly and are susceptible to other liver diseases, which may cause metabolic dysregulation, reduce growth performance, and impair both bone development and the oxidative response [9,10]. This disease has resulted in severe economic losses and become a serious risk to the GIFT aquaculture industry. A reduction in lipid deposition, increase in metabolites associated with β-oxidation, and reduction in fatty acid levels in the liver have been shown to improve the fatty liver condition. The molecular mechanism underlying the development of hepatic steatosis in GIFT with various diets remains largely unknown.

Carnitine acetyltransferase (CAT) as a key enzyme in energy metabolism transfers activated acyl CoA and carnitine on the outer membrane of mitochondria into acyl-carnitine and then into the mitochondrial matrix [11,12]. It mainly catalyzes the conversion of acyl groups between carnitine and coenzyme A [13] and is essential for the intracellular trafficking of fatty acids and fatty acid oxidation [14]. CAT localized in mitochondria, endoplasmic reticulum, and peroxisome [14,15] can modulate mitochondrial acetyl-CoA/CoA (coenzyme A) ratios, thus regulating pyruvate dehydrogenase activity and glucose oxidation [16,17]. To date, more studies have been conducted on the CAT gene in yeast, mice, and pigeons, but fewer studies have been conducted on the fish gene. The full-length cDNA encoding CPT I has been cloned and sequenced in several organisms, such as large yellow croaker (*Larimichthys crocea*) [18], yellow catfish (*Pelteobagrus fulvidraco*) [19], and Blunt snout bream (*Megalobrama amblycephala*) [20]. However, there is no report on the CAT gene of GIFT.

Mitochondrial fatty acid β-oxidation (FAO) is the primary pathway for fatty acid metabolism in humans, performing a key role in liver, heart, and skeletal muscle energy homeostasis. FAO is particularly important during times of fasting when glucose supply is limited, providing energy for many organs and tissues, including the heart, liver, and brain [21]. Short-chain enoyl-CoA hydratase (ECHS1), a mitochondrial matrix enzyme that catalyzes the second step of FAO [22,23], involves the hydration of chain-shortened α,β-unsaturated enoyl-CoA thioesters to produce β-hydroxyacyl-CoA [23], and release one acetyl-CoA molecule, which is utilized for either the formation of the TCA cycle, or to provide energy for the body’s metabolism [24,25]. ECHS1 deficiency can lead to fat deposition and disease [26,27]. Research on the expression and biochemical function of ECHS1 in GIFT is an important and urgent problem to solve. Carnitine and lipid diet were reported to play an important role in lipid metabolism [28,29]; however, there are no reports on the expression of CAT and ECHS1 in GIFT with different carnitine and fat levels. 

In this study, the cDNAs of CAT and ECHS1 genes were cloned and analyzed via bioinformatics, and the expressions of CAT and ECHS1 in the liver of juvenile GIFT were detected in different fat and carnitine diets, as were the changes in lipometabolic enzymes and serum biochemical indexes of juvenile GIFT in different fat and carnitine diets. We aimed to clarify the sequence and expression of two enzymes in GIFT liver with different fat and carnitine feeds, as well as the optimal dietary fat and carnitine supplemental levels, so as to provide a theoretical basis for GIFT feeding and the treatment of fatty liver disease.

## 2. Materials and Methods

### 2.1. Experimental Fish

In total, 1350 GIFT tilapia fries were obtained from the Guangxi Academy of Fishery Sciences. The total initial average weight of fish was 2.9 ± 0.3 g, and the initial average body length was 2.96 ± 0.03 cm. The experimental fish were domesticated with basic feed, adding different fats and different carnitines (Table 1). In accordance with to the feeding conditions, fish were randomly assigned into 9 groups, each group containing 3 duplicate groups with 50 fish per group (tank size: 1 m × 1 m × 1 m). During the feeding trial (70 d), fish were fed twice a day (9 a.m. and 6 p.m.), and satiated feeding was ensured. Fish wastes and half of the water were exchanged daily and replaced with well-aerated tap water. The experimental water was of a temperature of 26.9 ± 1.2 °C, at pH 6.8 ± 0.1, and had a dissolved oxygen level of 7.13 ± 0.15 mg/L. All the experiments were conducted using a protocol approved by Guangxi University (GXU-2022-249).

### 2.2. Sample Collection

All the fish were fasted for 24 h and then sampled. In total, 15 fish were randomly selected from each parallel group, placed in water containing 80 mg/L MS-222, and anesthetized. Blood was drawn from the caudal vein of the fish, left to stand at 4 °C for 4 h, and centrifuged at 4000 r for 10 min; the supernatant was serum, which was stored at −80 °C. After blood sampling, the fish were placed on ice for rapid dissection, and the liver tissue was removed, quick-frozen in liquid nitrogen, and stored at −80 °C. 

### 2.3. Determination of Serum Biochemical Indexes and Metabolic Enzymes

The serum obtained was used for the determination of ALT (alanine aminotransferase) (U/L), AST (aspartate aminotransferase) (U/L), LDH (lactate dehydrogenase) (U/L), CHOL (cholesterol) (mmol/L), TG (triglycerides) (mmol/L), HDL-C (high density lipoprotein cholesterol) (mmol/L), and LDL-C (low density lipoprotein cholesterol) (mmol/L) levels. Experimental methods were performed in accordance with the kit’s instructions (Nanjing Jiancheng, Nanjing, China). Frozen liver tissue was taken for tissue homogenization, and the supernatant was taken and centrifuged at 4 °C, 2500 r, for 20 min and stored at −80 °C for a metabolic enzyme activity assay. The activities of CACT (carnitine acyl transferase) (U/L), ACC (Acetyl coenzyme A carboxylase) (U/L), FAS (fatty acid synthetase) (nmol/L), HL (hepatic lipase) (μmol/L). and LPL (lipoprotein lipase) (U/L) were determined using ELISA Kit (Ze Yu, Yancheng, China).

### 2.4. RNA Isolation and cDNA Cloning

Total RNA from the liver of fish was determined using the Total RNA Extraction Kit (TaKaRa Code No: 9767). RNA integrity was verified via 1% (*w*/*v*) agarose gel electrophoresis, while the concentrations and purities were examined with a micro-ultraviolet spectrophotometer. Fast Quant First-Strand cDNA Synthesis Kit (TIANGEN, Beijing, China) was used to reverse-transcribe the first-strand cDNA, in accordance with the manufacturer’s instructions. The specific primers were designed using Primer Premier 5 and Oligo 6.0 software based on the sequences of the CAT gene and ECHS1 gene of Nile tilapia in the National Center for Biotechnology and Information (NCBI) (Table 2). Based on the intermediate fragments, 5′RACE- and 3′RACE-nested PCR-specific primers for CAT and ECHS1 genes were designed (Table 3). The purified DNA was cloned into the pMD18-T Vector (TaKaRa, Shiga, Japan), and then transformed into *Escherichia coli* DH5α-competent cells (TaKaRa, Shiga, Japan). Positive clones were selected and sent to biotechnology company (Lifei Biotechnology Co., Shanghai, China) for full-length sequencing.

The DNA copy number was calculated using the following formula:

$$No.of\;copies=\frac{6.02\cdot 10^{23}(copies\cdot {mol}^{-1})\cdot DNA\;amount(ng)}{DNA\;length(bp)\cdot 660(daltons\cdot bp-1)}$$

### 2.5. Sequence and Phylogenetic Analyses of CAT and ECHS1

Sequence splicing using SeqMan in DNASTAR 7.1 software, and the open reading frame (ORF), was analyzed with the ORF Finder (http://www.ncbi.nlm.nih.gov/gorf/gorf.html (10 March 2023)). BioEdit was used to deduce the encoded amino acid sequence, and the BLAST software of the NCBI was used to search the homologous genes and analyze their homology (NCBI, http://blast.ncbi.nlm.nih.gov/Blast.cgi (10 March 2023)). Multiple sequence alignments were performed using DNAman. The composition of amino acids was analyzed using the ExPASy—Prot Param tool. The online software of SignalP 4.1 Server (http://www.cbs.dtu.dk/services/SignalP/ (10 March 2023)) was used to detect the signal peptide of tilapia CAT and ECHS1 protein. Transmembrane structure was predicted using the online software TMHMM Server 2.0 (http://www.cbs.dtu.dk/services/TMHMM/ (10 March 2023)), and the SMART (http://smart.emblheidelberg.de/ (10 March 2023)) software was used to predict the protein domain features. The online software PSORT II was used for subcellular localization prediction. The secondary structure of protein was predicted using the online software of ORG (https://npsa-prabi.ibcp.fr/cgi-bin/npsa_automat.pl?page=npsa_gor4.html (10 March 2023)).

### 2.6. Real-Time Fluorescent Quantitative PCR (qRT-PCR)

The mRNA expression levels of CAT and ECHS1 were determined via qRT-PCR. In accordance with the full-length cDNA sequences of CAT and ECHS1 of the cloned GIFT, primers were designed for qRT-PCR using Primer Premier 5.0 (Table 4). 

qRT-PCR analysis for genes was performed by mixing 1 μL of cDNA template (100 µg/µL), 0.8 μL (10 μM) of each primer specific to genes, 10 μL of 2 × SYBR Premix Ex TaqⅡ, and 7.4 μL of RNase-free ddH_2_O. PCR reaction conditions were as follows: 95 °C for 30 s, ramp rate 4.4 °C/s; 40 cycles of 95 °C for 5 s, ramp rate 4.4 °C/s, 60 °C for 30 s, ramp rate 2.2 °C/s, and 60 °C~95 °C dissolution, increasing by 1 °C per 5 s. The melt curve was plotted at the end of the reaction to verify the specificity of the reaction system. The mRNA levels of target genes were calculated of based on the quantification cycle (Cq) value through the standard curve.

For the establishment of the standard curve, the concentrations of the standard plasmids of CAT and ECHS1 were 317.51 ng/uL and 203.06 ng/uL, respectively, as determined using an ultraviolet spectrophotometer. CAT and ECHS1 standard plasmids were serially diluted 10-fold into 5 concentration gradients, and blank controls were set for each concentration standard, with three replications per sample. qRT-PCR was performed using LightCycler^®^480. After the reaction is completed, the system automatically generates a standard curve with Lg (the copy number) as the X axis and the Ct cycle value for the Y axis, and each concentration of the plasmid standard falls on the corresponding position of the curve. The linear regression equations for the standard curves of CAT and ECSH1 are as follows: y = −3.4183x + 32.694, R^2^ = 0.9994; y = −3.6671x + 33.64, R^2^ = 0.9991. R^2^ > 0.99 of regression equations indicated that the linear relationship is extremely strong, and this standard curve can be used to quantify genes.

### 2.7. Statistical Analysis

A two-way ANOVA and Tukey’s multiple comparisons test were performed with Statistics Package for Social Science 24.0 software (SPSS, Inc., Chicago, IL, USA), and data are expressed as mean ± standard deviation (SD). *p* < 0.05 indicates a significant difference.

## 3. Results

### 3.1. Analysis of CAT and ECHS1 Gene Sequence and Deduced Protein

**Figure 1:**
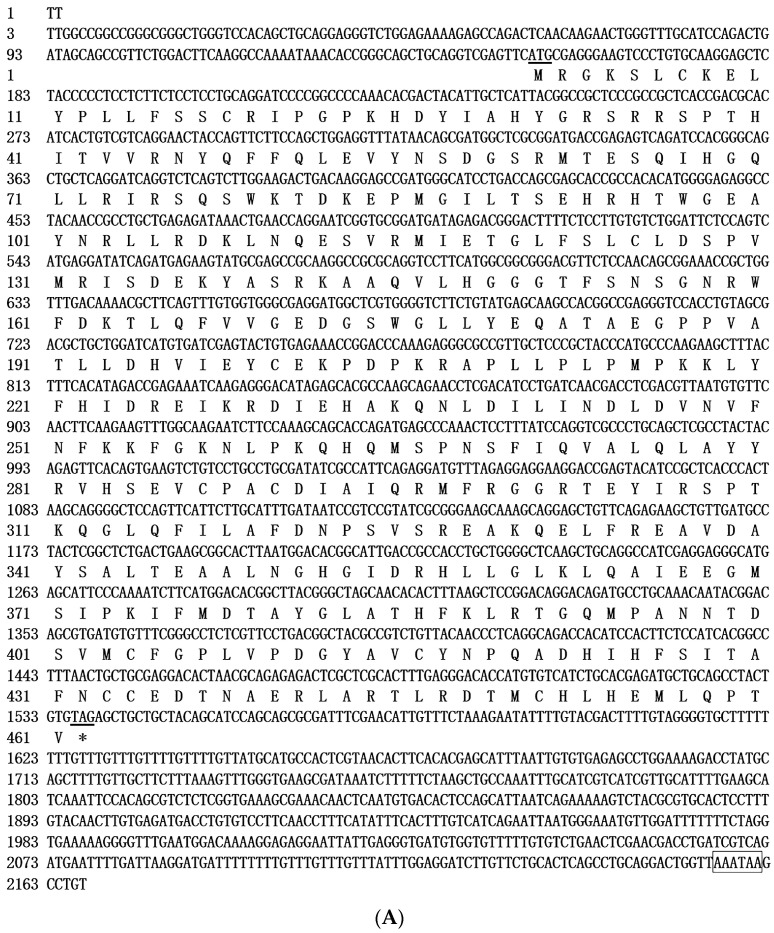

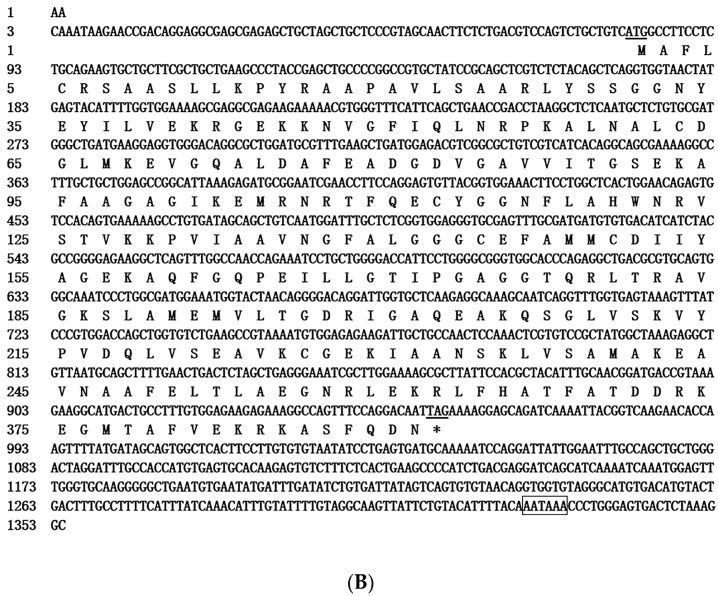
(**A**). Nucleotide and deduced amino acid sequences of GIFT CAT. The polyadenylation signal (AATAAA) in the 3′-UTR is boxed. The initiation codon (ATG) and termination codon (TAG) are underlined. (**B**). Nucleotide and deduced amino acid sequences of GIFT ECHS1. The polyadenylation signal (AATAAA) in the 3′-UTR is boxed. The initiation codon (ATG) and termination codon (TAG) are underlined. * represents the stop codon sequence.

The full-length sequence of CAT cDNA in GIFT was 2167 bp, and the ORF was 1383 bp; it encoded 461 amino acids. The 5′ noncoding region and 3′ noncoding region were 152 bp and 632 bp, respectively, and in the 3′ noncoding region existed the polyadenylation signal (AATAAA), demonstrating that the obtained fragment included a full-length mRNA 3′ non-coding region (Figure 1A). The molecular weight of CAT protein was estimated to be 52.6 kDa. The area -MSPNSFIQVALQLAYYR-DAIQRMFRGGRTE-RS-GHGIDRHLLGLKLQAI in the CAT sequence contains the Arg, His, Tyr, Thr, and other active amino acid residues, and CAT proteins have an Arg conserved domain with a ligand-binding domain for carnitine and acyl-CoA [13,30], and at least three different active domains [31,32]. The domain prediction demonstrated that CAT protein contains carnitine acyl transferase activity domains, consistent with the findings of Holden et al. [32]. Bioinformatics analysis also showed that CAT was free of the signal peptide, and subcellular localization in the cytoplasm suggested that CAT is a non-secreted protein. Protein secondary structure analysis showed that CAT consists of an α helix, extended strand, and random coil with a ratio of 37.53%, 15.62%, and 46.85%, respectively.

The ECHS1 cDNA sequence was 1354 bp in full length, the ORF of which encoded a peptide of 391 amino acids with a molecul0ar weight of 31.08 kDa (Figure 1B). The length of the 5′ noncoding region and 3′ noncoding was 80 bp and 101 bp, respectively. ECHS1 amino acid sequence contains conserved amino acid sequences, such as -GGGCEFAMMCDIIYAGEKAQFGQPEILLGTIPGAGGTQRLTTRAVGKSLAMEMVLTGDRI, in which Gly and Glu are included, and this is consistent with the views of Agnihotri et al. [33] and He et al. [34]. The enoyl coenzyme A hydratase active domain was found in the ECHS1 sequence. Bioinformatics analysis also showed that ECHS1 is a non-secreted protein and is free of a signal peptide, and the transmembrane domain was found in the 129–149 amino acid sequence of the ECHS1 transmembrane region. Similar to CAT, the secondary structure of ECHS1 was made up of 48.45% α helix, 19.17% extended strand, and 34.36% random coil.

### 3.2. Multiple-Sequence Alignment and Phylogenetic Analysis of CAT and ECHS1 Gene

**Figure 2:**
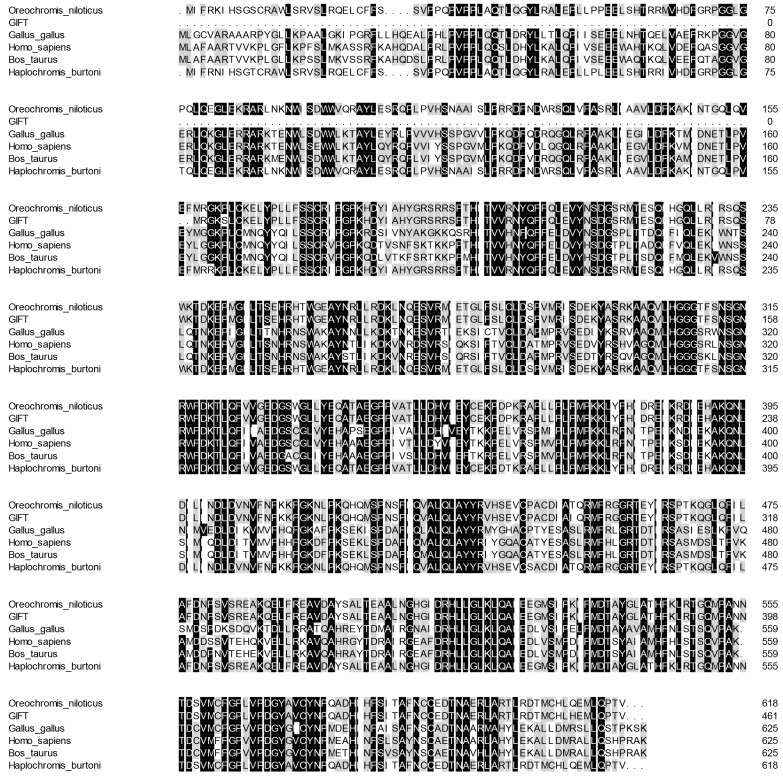
Multiple alignments of GIFT CAT with other known CAT deduced amino acid sequences: *O. niloticus* (XM_005457163.4), *H*. *burtoni* (XM_042222673.1), *Gallus gallus* (NM_001031215.2), *Homo sapiens* (NM_001752.4), and *Bos taurus* (NM_001035386.2). Dark colors represent identical amino acids, light colors represent similar amino acids.

BLAST analysis showed that the deduced amino acid sequence of CAT in GIFT was aligned (54~99%) with other previously reported CAT proteins. According to the sequence alignment results, the amino acid sequence of CAT in GIFT shared high similarity (above 98%) with its homologs from other teleosts including Nile tilapia (*O. niloticus*) and Burton’s mouthbrooder (*Haplochromis burtoni*) (Figure 2).

**Figure 3:**
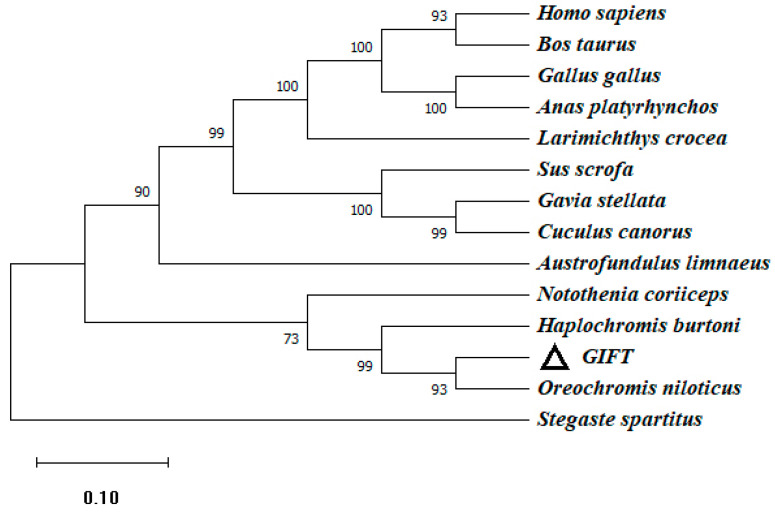
The phylogenetic tree based on the amino acid sequences of the CAT gene of GIFT and other species. This is a neighbor-joining phylogenetic tree of the CAT mature peptide from GIFT and other vertebrates. The evolutionary tree was constructed using MEGA 11.0 software. The percentage of replicate trees in which the associated taxa clustered together in the bootstrap test (1000 replicates) is shown next to the branches. The accession numbers of *O. niloticus* (XM_005457163.4), *H*. *burtoni* (XM_042222673.1), *Homo_sapiens* (NM_001752.4), *Notothenia_coriiceps* (XM_010780775.1), *Gavia_stellata* (XM_059828778.1), *Sus_scrofa* (XM_005660485.3), *Stegaste_spartitus* (XM_008291538.1), *Austrofundulus_limnaeus* (XM_014013093.1), *Bos_taurus* (NM_001035386.2), *Gallus_gallus* (NM_001031215.2), *Anas_platyrhynchos* (XM_027458335.2), *Larimichthys_crocea* (XM_010735178.3), and *Cuculus_canorus* (XM_054068522.1) are shown. Numbers in the phylogenetic tree represent branching confidence and scales represent genetic variability.

To investigate the genetic characteristics and phylogenetic relationships, we further analyzed the phylogenetic trees of CATs in different species. An NJ phylogenetic tree was constructed, within the fish CAT cluster, and GIFT shared a closer relationship with Nile tilapia and Burton’s mouthbrooder (Figure 3).

**Figure 4:**
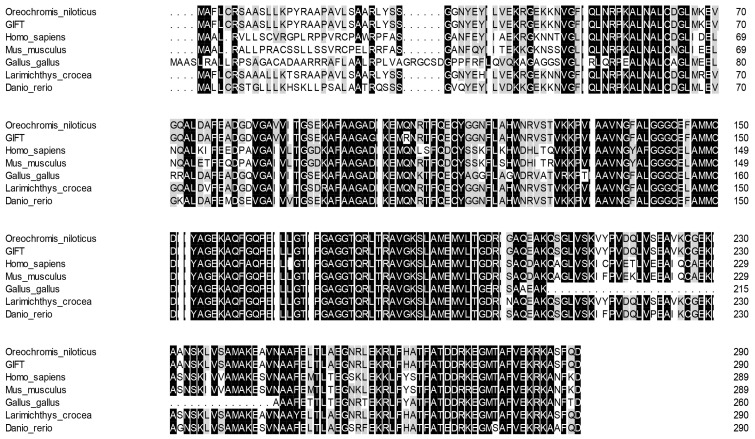
Multiple alignments of GIFT ECHS1 with other known ECHS1 deduced amino acid sequences: *O. niloticus* (XM_003441181.5), *Mus musculus* (XM_011249839.4), *L. crocea* (XM_019259592.2), *H. sapiens* (NM_004092.4), *G. gallus* (XM_046942973.1), and *D. rerio* (NM_001004529.2). Dark colors represent identical amino acids, light colors represent similar amino acids.

**Figure 5:**
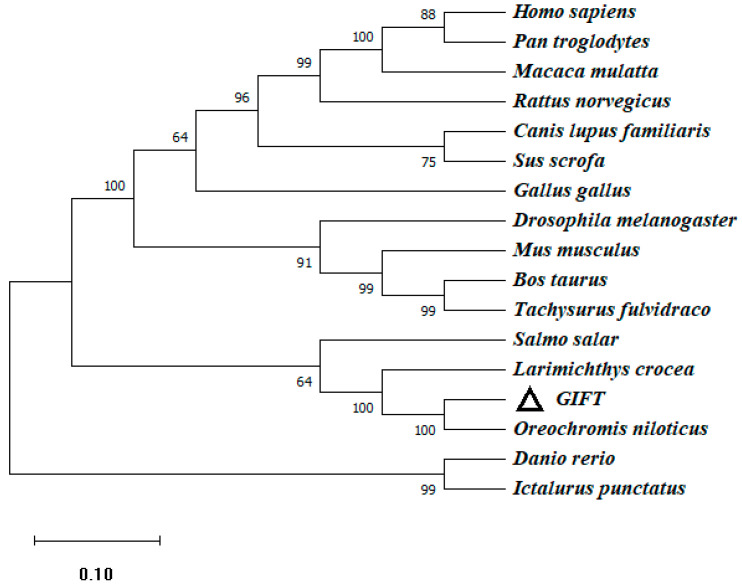
The phylogenetic tree (NJ) based on the amino acid sequences of the ECHS1 gene of GIFT and other species. This is a neighbor-joining phylogenetic tree of the ECHS1 mature peptide from GIFT and other vertebrates. The tree was constructed using MEGA 11.0 software. Aligned sequences were bootstrapped 1000 times. The accession numbers of *O. niloticus* (XM_003441181.5), *L. crocea* (XM_019259592.2), *H. sapiens* (NM_004092.4), *Drosophila melanogaster* (NM_137066.4), *Rattus norvegicus* (NM_078623.2), *M. musculus* (XM_011249839.4), *D. rerio* (NM_001004529.2), *B. taurus* (NM_001389968.1), *Canis lupus familiaris* (XM_038441020.1), *G. gallus* (XM_046942973.1), *Pan troglodytes* (XM_016919678.4), *Sus scrofa* (NM_001190175.1), *Macaca mulatta* (XM_015148517.2), *Salmo salar* (NM_001139656.1), *Ictalurus punctatus* (NM_001201117.1), and *Tachysurus fulvidraco* (XM_027134599.2) are shown. Numbers in the phylogenetic tree represent branching confidence and scales represent genetic variability.

The similarity of ECHS1 of the GIFT amino acid sequence between different species was consistent (66~99%) with other similarities observed using BLAST analysis. According to the sequence alignment results, the ECHS1 protein sequence shared high similarity (above 85%) with its homologs from other teleosts including Nile tilapia (*O. niloticus*), large yellow croaker (*L*. *crocea*), and zebrafish (*Danio rerio*) (Figure 4). Phylogenetic analysis based on ECHS1 sequences showed that GIFT ECHS1 was closest to Nile tilapia ECHS1, with 99% similarity, and had the highest similarity to ECHS1 of large yellow croaker in the fish species (Figure 5).

### 3.3. Expression Level of CAT Gene in Juvenile GIFT Liver within Different Fat and Carnitine Diets

**Figure 6:**
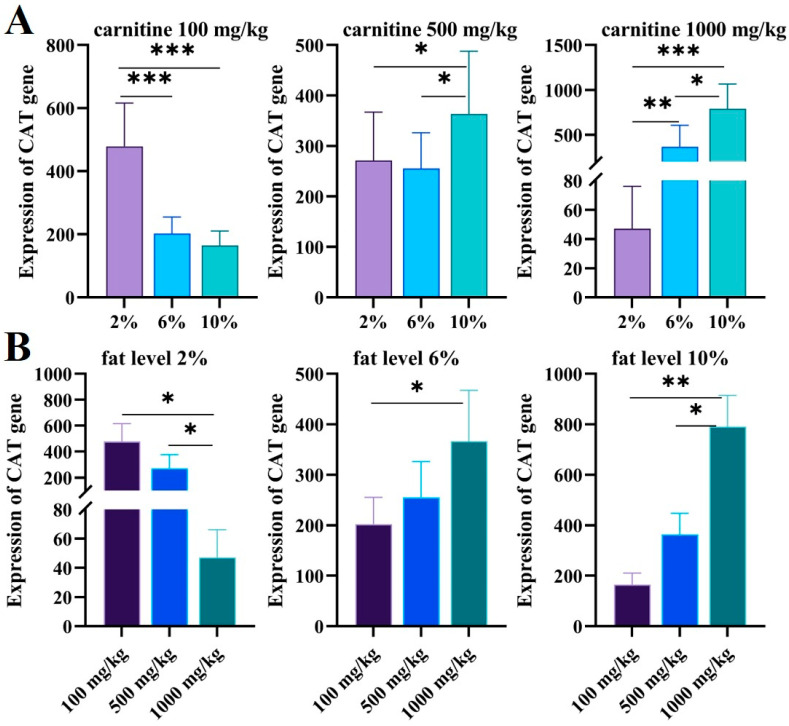
(**A**) The expression levels of CAT in different carnitine feeds in GIFT livers with increasing fat levels were validated via qRT-PCR. The X axis represents the fat level and the Y axis represents the expression of CAT genes. (**B**) The expression levels of CAT in different fat feeds for GIFT livers with increasing carnitine levels were validated via qRT-PCR. The X axis represents carnitine level and Y axis represents expression of CAT genes. The expression of CAT genes was valued via the CAT standard curve. Data are expressed as mean ± SD (*, *p* < 0.05; **, *p* < 0.01; ***, *p* < 0.001).

After feeding juvenile GIFT with different carnitine and fat diets, we found that the expression of CAT in the juveniles was different. As shown in Figure 6A, when the carnitine level was 100 mg/kg, the expression of the CAT gene decreased significantly with the increase in feed fat (*p* < 0.05). When the carnitine concentration was 500 mg/kg, the expression level of the CAT gene was not significantly affected by the feed’s low levels of fat (2% and 6%) (*p* > 0.05). Interestingly, when the carnitine concentration was 1000 mg/kg, the expression of the CAT gene increased significantly with increasing fat levels (*p* < 0.05). 

When the fat level was 2%, the expression of the CAT gene decreased significantly with the increase in carnitine levels (*p* < 0.05); when the fat level was 6% and 10%, the expression of the CAT gene increased with the increase in carnitine levels (*p* < 0.05) (Figure 6B). 

### 3.4. Expression Level of ECHS1 in Juvenile GIFT Liver within Different Fat and Carnitine Diets

**Figure 7:**
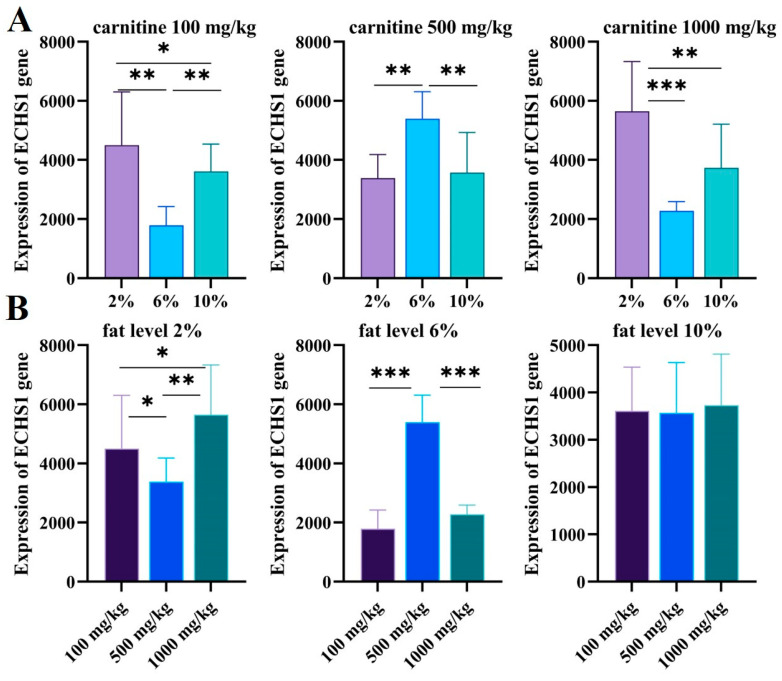
(**A**) The expression levels of ECHS1 in different carnitine feeds for GIFT livers with increasing fat levels were validated via qRT-PCR. The X axis represents the fat level and the Y axis represents the expression of ECHS1 genes. (**B**) The expression levels of ECHS1 in different-level fat feeds for GIFT livers with increasing carnitine levels were validated via qRT-PCR. The X axis represents the carnitine level and the Y axis represents the expression of ECHS1 genes. The expression of CAT genes was valued based on the ECHS1 standard curve. Data are expressed as mean ± SD (*, *p* < 0.05; **, *p* < 0.01; ***, *p* < 0.001).

With different fat and carnitine diets, the transcript levels of ECHS1 in the liver of GIFT were different. We found that the 6% fat level group was significantly different from the 2% and 10% fat level groups (Figure 7A); ECHS1 in the 2% and 10% fat level groups was higher than ECHS1 in the 6% fat level group when the concentration of carnitine was 100 mg/kg and 1000 mg/kg, and the opposite trend was found for 500 mg/kg feeds.

There was no significant difference in ECHS1 levels when the fat level was 10% (*p* > 0.05), and the expression of ECHS1 was significantly affected by the 2% and 6% fat levels. When the feed fat level was 2%, the expression level of the ECHS1 gene decreased significantly with 500 mg/kg carnitine feeding (*p* < 0.05) and then increased significantly with 1000 mg/kg carnitine feeding (*p* < 0.01); the amount of carnitine of 500 mg/kg had the lowest CAT level. At the 6% fat level, the expression level of the ECHS1 gene increased significantly with the increasing carnitine level (*p* < 0.001), then decreased significantly (*p* < 0.001), and reached the peak when the amount of carnitine was 500 mg/kg (Figure 7B).

### 3.5. Effects of Dietary Fat and Carnitine Levels on Blood Biochemical Indicators and Metabolic Enzyme Activities of GIFT

As can be seen from Table 5, the activities of CACT, ACC, FAS, HL, and LPL of tilapia were significantly affected by the fat level and carnitine level (*p* < 0.05). The interaction of fat and carnitine with CACT, ACC, FAS, HL, and LPL and the differences between groups were extremely significant (*p* < 0.01). CACT activity increased and then decreased with increasing carnitine levels at 2% and 10% fat levels. The lowest ACC activity of 175.08 ± 8.30 was observed at a 10% fat level and 1000 mg/kg carnitine level. The activity of FAS showed a decreasing trend under the influence of fat and carnitine. HL activity was higher at a 10% fat level and at 500 mg/kg and 1000 mg/kg carnitine levels. The highest LPL activity of 175.08 ± 8.30 was observed at a 2% fat level and 100 mg/kg carnitine level.

As can be seen from Table 6, under the different fat level and carnitine level conditions, ALT, AST, and LDH of GIFT tilapia were significantly different (*p* < 0.05). The interactions of ALT, AST, and LDH and the differences between groups were significantly affected by fat and carnitine levels (*p* < 0.01). When the fat level was the same, the activities of ALT, AST, and LDH showed a trend of decreasing first and then increasing slowly with the increase in carnitine levels. The group with 6% fat and 500 mg/kg carnitine had the lowest ALT activity (15.67 ± 3.79). AST and LDH activities were lowest at a 2% fat level and 100 mg/kg carnitine level, with values of 98.33 ± 13.61 and 488.67 ± 8.74, respectively.

As can be seen from Table 6, there were no significant differences in the effects of the fat level on CHOL, TG, HDL-C, and LDL-C of GIFT tilapia (*p* > 0.05). The effects of carnitine on CHOL, TG, HDL-C, and LDL-C were significantly different (*p* < 0.01). The interaction of fat and carnitine levels with TG was significant (*p* < 0.05), but the interaction of CHOL, HDL-C, and LDL-C was not significant (*p* > 0.05). Fat and carnitine had significant effects on CHOL, TG, and HDL-C among groups (*p* < 0.05), but had no significant effects on LDL-C among groups (*p* > 0.05). When the fat level was the same, the content of CHOL firstly decreased and then increased with the increase in carnitine levels. The group with a 6% fat level and 100 mg/kg carnitine level had the highest value (2.29 ± 0.44). With the increase in fat and carnitine contents, TG content, on the whole, showed a tendency of slow decrease first and then increase, and the group with a 6% fat and 500 mg/kg carnitine level had the lowest value (0.31 ± 0.02). With the increase in fat and carnitine levels, the content of HDL-C showed a trend of slow decrease at first and then increase, and the group with higher fat levels had higher HDL-C levels overall. LDL-C content was lower in the group with a carnitine level of 500 mg/kg.

## 4. Discussion

Hepatic steatosis, a common disease in humans and animals, is characterized by imbalanced lipid metabolism and the accumulation of triglycerides in hepatocytes, and has become one of the serious concerns to the aquaculture industry [35,36]. However, little information is available about the underlying molecular mechanisms of hepatic lipid metabolism in tilapia. CAT and ECHS1 were reported to be important enzymes in the fat metabolism of fatty liver disease. To investigate the whole length and expression of CAT and ECHS1 in different lipid and carnitine levels, we obtained the cDNA of CAT and ECHS1 and analyzed the genetic information and conducted protein property judgment and prediction via sequencing. We confirmed that the full-length sequence of CAT cDNA was 2167 bp, and the ORF was 1383 bpl it encoded a total of 461 amino acids. The ECHS1 cDNA sequence was 1354 bp in full length, and the ORF encoded a peptide of 391 amino acids. When different carnitine and fat diets were given to juvenile GIFT, the effect on the expression between CAT and ECHS1 was different as well. 

Increasing fat levels will inhibit the expression of fatty acid synthase in the liver of fish [37]. Oxidized oil feeding may inhibit fat metabolism enzymes and affect the synthesis and homeostasis of cholesterol and fatty acids [38]. Conversely, feeding mice with a high-fat diet increased the cardiac CAT protein level and its activity, which is associated with a reduced acetyl-CoA/CoA ratio and glucose oxidation [39]. In this experiment, when the carnitine content was 100 mg/kg, the expression of the CAT gene decreased with the increasing levels of fat in the feed, and when the carnitine level was 1000 mg/kg, the expression level of the CAT gene increased significantly with the increasing fat level. This may be related to the acceleration of the metabolic reaction of fat when the body’s intake of fat is increased, and may also be related to the reduction in body fat deposition by carnitine. L-carnitine can also affect the digestion and absorption of fatty acids [40]. However, L-carnitine has no significant effect on reducing fat deposition in carp [41]. We found that when the fat level was 6% and 10%, the expression of the CAT gene increased significantly with the increasing carnitine levels, which was consistent with the research view for Karlic Hon rats [42]. This may be related to the conversion of CAT, primarily catalyzing the conversion of acyl groups between carnitine and CoA. When the fat levels were 2%, the expression of the CAT gene decreased with the increase in carnitine levels, which may be related to the promotion of LPL and PPAR gene expression at low energy levels. It may also be related to the low level of fatty acid metabolism at low energy levels, and high fat levels inhibit the antioxidant capacity of tilapia [43].

Mitochondrial fatty acid β-oxidation is an essential pathway in providing energy [44]. ECHS1 is a mitochondrial enzyme involved in the oxidation of fatty acids and the catabolic pathway of valine [24]. The passage of medium-chain fatty acyl-CoA esters through the mitochondrial membrane is facilitated by the L-carnitine system [45]. Research on ECHS1 expression related to different fat and carnitine diets is still unclear. This study found that the expression of ECHS1 was not significantly different with the increase in carnitine levels in feed at a 10% fat level, which may be related to the decrease in fatty acid metabolism enzyme activity at high fat levels, and ECHS1 expression was closely associated with fat and carnitine feeding. 

CACT is a family of enzymes that selectively catalyze substrates of different carbon chain lengths and play an indispensable role in the decomposition of fatty acids [46]. ACC requires seven enzymatic reactions before it can be synthesized into palmitic acid. The above steps are important for the synthesis of fatty acids. FAS is a complex enzyme that controls the amount of fat synthesis in the body through its activity. Previous studies have found that the higher the dietary fat content of grouper (*Epinephelus coioides*), the higher the lipase activity. In this experiment, when the fat level increased from 2% to 6%, the CACT and HL activities of tilapia increased with the increase of in fat level. Barahi et al. [47] studied the lipometabolic enzymes of Morone Saxatilis, and Pedersen et al. [48] studied the lipometabolic enzymes of *Clupea pallasi* juvenile fish; they found that the activity of lipase in fish was affected by the different natures of food intake. When fat levels were consistent, the activities of FAS and LPL decreased with the increase in carnitine levels. The lipoprotein’s rough endoplasmic reticulum is synthesized in liver cells, combined with liver lipids to be secreted into the cytoplasm through the Golgi apparatus, and then combined with the blood in the liver to balance the metabolism of fat and blood lipids in the liver. When the amount of lipoprotein is synthesized, the liver fat in the cell cannot be transported in time, which will cause the fat to accumulate in the liver. When the dietary fat level was 10%, the HL activity of tilapia showed a trend of firstly increasing and then slowly decreasing. 

ALT and AST are important aminotransferases, and protein metabolism is mainly controlled by them. The intensity of amino acid metabolism and the degree of liver damage can be reflected by the activity of ALT and AST in the liver and pancreas. In this experiment, when the level of carnitine was the same, the activity of ALT and AST decreased first and then slowly increased with the increase in fat levels. This may have been because the increased amount of fat fed caused liver tissue lesions. Intracellular ALT and AST are released into the blood, but because of the presence of carnitine, the two form an antagonistic effect, which leads to a slow increase in the ALT and AST activities in the serum biochemical indicators. When the amount of LDH is appropriate, it shows an upward trend with the increase in fat levels. In this test, when the fat level was increased from 2% to 6%, the LDH of GIFT increased with the increase in the fat level, but when it was 10%, the vitality of GIFT’s LDH decreased, and the difference was extremely significant (*p* < 0.01). The researchers administered L-carnitine to mice, and the results showed that carnitine can significantly extend the weight-bearing swimming time of mice. After swimming fatigue, the lactic acid levels in mice do not increase, which is in accordance with the results shown in this experiment.

TG and CHOL are the main lipids in the blood. They operate between various tissues and can reflect the lipid metabolism in the body [49]. Fish cholesterol mainly comes from the synthesis of the liver, and the rest comes from the digestive tract. If liver disease occurs, the level of CHOL in the blood will increase. In this experiment, as the feed fat level increased, the CHOL concentration also increased. When the fat level was 10%, the CHOL concentration increased significantly, and the increase in CHOL was not conducive to the growth of fish. When the amount of feed carnitine was 500 mg/kg, the concentration of CHOL increased significantly with the increase in fat levels; when the fat level reached 10%, it had already caused damage to the animal liver, so high levels of carnitine cannot reverse the damage that high levels of fat cause to the liver. Due to the physiological functions of carnitine, it can remove free acyl groups in the body. The main raw material for the liver to synthesize CHOL with is acetyl CoA, so the added free L-carnitine is absorbed and combined with the acyl group in the body, which reduces the amount of raw materials for the liver to synthesize CHOL with, thereby reducing the synthesis of CHOL in the body. A decrease in the synthesis of CHOL in the body will reduce the CHOL in the blood, which shows that L-carnitine plays a certain role in the metabolism of CHOL.

Lin et al. [50] showed that the fat content of grass carp suffering from fatty liver disease is negatively correlated with the serum TG content. In the experiment, as the fat content in the feed increased, the TG content first decreased, and the change trend showed a negative correlation. When the carnitine level was increased to 500 mg/kg, as the fat level increased, the tissue in the liver was destroyed, and the HDL-C content in the blood increased. This situation should be related to the addition of carnitine to the feed, indicating that carnitine can effectively eliminate the fat accumulated in the liver. Previous studies have shown that if LDL-C transport has a reduced ability to remove exogenous fats, it can lead to a decrease in TG levels, which in turn leads to a decrease in LDL-C in the blood. In our study, when the carnitine level was 100 mg/kg, the LDL-C content decreased as the fat levels increased. When carnitine levels exceeded 500 mg/kg, the LDL-C content of GIFT showed an inverse trend. 

## 5. Conclusions

In our present study, the full-length and ORF sequence of CAT and ECHS1 genes in GIFT liver were obtained. We found that GIFT had higher lipometabolic enzyme activity and lower blood CHOL, TG, HDL-C, and LDL-C contents when the dietary fat level was 2% or 6% and when the carnitine level was 500 mg/kg. We also found that the expression of ECHS1 and CAT genes in the liver of GIFT can be promoted by 500 mg/kg carnitine level and 6% fat level feeding. These results suggested that CAT and ECHS1 may participate in the regulation of lipid metabolism, and when 2% or 6% fat and 500 mg/kg of carnitine are added to the feed, it is most beneficial to the liver and lipid metabolism of GIFT.

## Figures and Tables

**Table 1:** Nutrient level and composition of experimental diet (%).

Material	Group								
	1	2	3	4	5	6	7	8	9
Fish meal	5	5	5	5	5	5	5	5	5
Soybean meal	43	43	43	43	43	43	43	43	43
Corn	15	15	15	15	15	15	15	15	15
Soybean	9	9	9	9	9	9	9	9	9
Rape meal	12	12	12	12	12	12	12	12	12
Calcium phosphate	1.5	1.5	1.5	1.5	1.5	1.5	1.5	1.5	1.5
NaCl	0.5	0.5	0.5	0.5	0.5	0.5	0.5	0.5	0.5
Choline Chloride	1	1	1	1	1	1	1	1	1
Vitamins premix	0.5	0.5	0.5	0.5	0.5	0.5	0.5	0.5	0.5
Mineral premix	0.25	0.25	0.25	0.25	0.25	0.25	0.25	0.25	0.25
Soybean oil	2	2	2	6	6	6	10	10	10
Carnitine	0.01	0.05	0.1	0.01	0.05	0.1	0.01	0.05	0.1
Carboxymethyl cellulose	2	2	2	2	2	2	2	2	2
Microcrystalline cellulose	8.24	8.2	8.15	4.24	4.2	4.15	0.24	0.2	0.15
Total	100	100	100	100	100	100	100	100	100
Main nutrients									
Crude protein	35.61	35.54	36.17	35.59	35.39	35.43	35.58	34.96	35.50
Crude fat	5.16	5.42	5.23	9.07	8.93	9.34	12.98	12.47	12.76
Moisture	7.00	7.25	7.02	7.15	7.17	7.43	7.22	7.39	7.45

**Table 2:** Primer sequences for cloning the middle segment of the CAT and ECHS1 gene in the liver of GIFT.

Primer	Sequences	Product Expected Length/bp	Tm/°C
CAT1-F	CTGATAGCAGCCGTTCTGGACTT	751	62.7
CAT1-R	CCTCTTGATTTCTCGGTCTATGTGA		61.9
CAT2-F	GGACATAGAGCACGCCAAGC	1297	60.6
CAT2-R	TGCAGAACAAGATCCTCCAAATA		59.3
ECHS1-F	CCAACAAATAAGAACCGACAGGA	863	61.1
ECHS1-R	TTTCCAAGCGATTTCCCTCA		60.6

**Table 3:** Primer sequences for the 5′RACE and 3′RACE cloning of the CAT and ECHS1 gene in the liver of GIFT.

Primer	Sequences	Product Expected Length/bp
5′RACE Outer Primer	CATGGCTACATGCTGACAGCCTA	23
5′RACE Inner Primer	CGCGGATCCACAGCCTACTGATGATCAGTCGATG	34
5′CAT-GSP1	CCCCAAACACGACTACATTGCTCAT	25
5′CAT-GSP2	ACACGACTACATTGCTCATTACGGC	25
5′ECHS1-GSP1	AGCCCATCGCACAGAGCATTGAG	23
5′ECHS1-GSP2	TCCCACCTCCTTCATCAGCCCAT	23
3′RACE Outer Primer	TACCGTCGTTCCACTAGTGATTT	23
3′RACE Inner Primer	CGCGGATCCTCCACTAGTGATTTCACTATAGG	32
3′CAT-GSP1	AGGTGAAAAAGGGGTTTGAATGG	23
3′CAT-GSP2	AGGGTGATGTGGTGTTTTTGTGT	23
3′ECHS1-GSP1	CAAATCCCTGGCGATGGAAATGG	23
3′ECHS1-GSP2	GGTGTCTGAAGCCGTAAAATGTGG	24

**Table 4:** Primers for qRT-PCR.

Primer	Sequences	Tm/°C
QT-CAT-F	CGCCTGCTGAGAGATAAACTGAA	63.05
QT-CAT-R	CGCCCACCACAAACTGAA	61.72
QT-ECHS1-F	AGGGGACAGGATTGGTGCT	61.87
QT-ECHS1-R	TTCTCTCCACATTTTACGGCTTC	61.77

**Table 5:** Effects of fat and carnitine levels on lipid metabolism enzymes in GIFT.

Fat Level (%BW/d)	Carnitine Level (mg·kg^−1^)	CACT (U/L)	ACC (U/L)	FAS (nmol/L)	HL (μmol/L)	LPL (U/L)
2%	100	74.27 ± 0.93 ^c^	185.83 ± 8.77 ^ab^	6.64 ± 0.17 ^f^	89.81 ± 1.97 ^c^	56.50 ± 1.26 ^d^
2%	500	77.03 ± 3.18 ^c^	218.74 ± 8.56 ^c^	5.28 ± 0.20 ^bcd^	61.97 ± 4.50 ^a^	47.31 ± 2.00 ^bc^
2%	1000	63.21 ± 0.93 ^b^	198.28 ± 6.08 ^b^	4.38 ± 0.10 ^a^	85.73 ± 4.74 ^c^	40.87 ± 1.99 ^a^
6%	100	98.54 ± 1.62 ^e^	180.56 ± 13.19 ^a^	6.50 ± 0.10 ^f^	106.24 ± 2.70 ^de^	55.13 ± 1.62 ^de^
6%	500	48.57 ± 1.69 ^a^	260.29 ± 6.24 ^d^	4.99 ± 0.26 ^c^	104.75 ± 1.28 ^d^	43.61 ± 1.66 ^ab^
6%	1000	65.38 ± 1.57 ^b^	308.59 ± 1.67 ^e^	5.66 ± 0.27 ^e^	76.17 ± 2.09 ^b^	48.57 ± 2.49 ^c^
10%	100	91.37 ± 2.54 ^d^	304.79 ± 4.56 ^e^	5.40 ± 0.12 ^de^	61.23 ± 2.75 ^a^	46.78 ± 2.56 ^bc^
10%	500	100.18 ± 3.60 ^e^	175.08 ± 8.30 ^a^	4.80 ± 0.14 ^b^	111.90 ± 2.70 ^f^	41.90 ± 2.84 ^a^
10%	1000	91.14 ± 3.18 ^d^	326.72 ± 4.31 ^f^	5.02 ± 0.23 ^bc^	110.60 ± 3.42 ^ef^	44.07 ± 4.24 ^abc^
*p*-values						
Fat level		0.000	0.000	0.000	0.000	0.002
Carnitine level		0.000	0.000	0.000	0.000	0.000
Fat level × Carnitine level		0.000	0.000	0.000	0.000	0.001

Note: Different letters in the superscript represent significant differences.

**Table 6:** Effects of fat and carnitine levels on serum biochemical index in GIFT.

Fat Level (%BW/d)	Carnitine Level (mg·kg^−1^)	ALT (U/L)	AST (U/L)	LDH (U/L)	CHOL (mmol/L)	TG (mmol/L)	HDL-C (mmol/L)	LDL-C (mmol/L)
2%	100	23.33 ± 3.06 ^ab^	189 ± 11.27 ^e^	1102.33 ± 70.00 ^d^	2.09 ± 0.49 ^b^	0.51 ± 0.20 ^a^	1.58 ± 0.27 ^c^	0.31 ± 0.13 ^bc^
2%	500	16.33 ± 3.06 ^a^	98.33 ± 13.61 ^a^	488.67 ± 8.74 ^a^	1.08 ± 0.09 ^a^	0.31 ± 0.08 ^a^	0.98 ± 0.09 ^a^	0.11 ± 0.01 ^a^
2%	1000	21.67 ± 7.02 ^ab^	105.33 ± 17.03 ^ab^	533 ± 31.10 ^a^	1.30 ± 0.50 ^a^	0.52 ± 0.20 ^a^	1.11 ± 0.34 ^ab^	0.15 ± 0.06 ^ab^
6%	100	53.67 ± 9.07 ^d^	283.33 ± 28.68 ^f^	2142.33 ± 165.67 ^e^	2.29 ± 0.44 ^b^	0.55 ± 0.09 ^a^	1.56 ± 0.29 ^c^	0.26 ± 0.09 ^abc^
6%	500	15.67 ± 3.79 ^a^	116.67 ± 10.07 ^abc^	871 ± 89.37 ^bc^	1.24 ± 0.27 ^a^	0.31 ± 0.02 ^a^	1.07 ± 0.22 ^ab^	0.15 ± 0.03 ^ab^
6%	1000	18.67 ± 3.79 ^ab^	135.67 ± 27.10 ^abcd^	939.67 ± 54.41 ^cd^	1.66 ± 0.57 ^ab^	0.38 ± 0.08 ^a^	1.35 ± 0.36 ^abc^	0.23 ± 0.13 ^abc^
10%	100	28.33 ± 7.37 ^b^	165.67 ± 29.00 ^de^	792.67 ± 196.29 ^bc^	1.73 ± 0.15 ^ab^	0.45 ± 0.03 ^a^	1.41 ± 0.08 ^bc^	0.2 ± 0.02 ^abc^
10%	500	23.67 ± 6.80 ^ab^	144.33 ± 21.73 ^bcd^	665.67 ± 114.41 ^ab^	1.64 ± 0.10 ^ab^	0.38 ± 0.08 ^a^	1.33 ± 0.04 ^abc^	0.18 ± 0.03 ^ab^
10%	1000	30.0 ± 7.21 ^b^	154 ± 28.35 ^cde^	854.67 ± 137.57 ^bc^	2.20 ± 0.46 ^b^	0.90 ± 0.25 ^b^	1.47 ± 0.15 ^bc^	0.35 ± 0.15 ^c^
*p*-values								
Fat level		0.015	0.001	0	0.152	0.051	0.291	0.435
Carnitine level		0	0	0	0.004	0.002	0.008	0.034
Fat level × Carnitine level		0	0	0	0.065	0.013	0.266	0.095

Note: Different letters in the superscript represent significant differences.

## Data Availability

All data generated or analyzed during this study are included in this published article.
